# A biological security motivation system for potential threats: are there implications for policy-making?

**DOI:** 10.3389/fnhum.2013.00556

**Published:** 2013-09-09

**Authors:** Erik Z. Woody, Henry Szechtman

**Affiliations:** ^1^Department of Psychology, University of WaterlooWaterloo, ON, Canada; ^2^Department of Psychiatry and Behavioural Neurosciences, McMaster UniversityHamilton, ON, Canada

**Keywords:** potential danger, precautionary behavior, security motivation, risk, decision making, obsessive-compulsive disorder (OCD)

## Abstract

Research indicates that there is a specially adapted, hard-wired brain circuit, the security motivation system, which evolved to manage potential threats, such as the possibility of contamination or predation. The existence of this system may have important implications for policy-making related to security. The system is sensitive to partial, uncertain cues of potential danger, detection of which activates a persistent, potent motivational state of wariness or anxiety. This state motivates behaviors to probe the potential danger, such as checking, and to correct for it, such as washing. Engagement in these behaviors serves as the terminating feedback for the activation of the system. Because security motivation theory makes predictions about what kinds of stimuli activate security motivation and what conditions terminate it, the theory may have applications both in understanding how policy-makers can best influence others, such as the public, and also in understanding the behavior of policy-makers themselves.

## Introduction

The world in which we currently live confronts people responsible for making decisions about security with very challenging issues. These issues call for sophisticated logical and statistical analysis, detection and forecasting systems, cost-benefit analysis, and the like. However, the crux of security is the necessity of dealing with the prospect of potential danger. Because potential dangers have had very substantial consequences for reproductive fitness for many thousands of years, evolution has shaped brain systems specially adapted for managing them. Thus, in addition to the logical armamentarium that present-day decision-makers bring to issues of security, they inevitably bring the intuitions and motivations that are generated by a biologically ancient, “hard-wired” system.

This potential-threat system in the brain has been termed the defense system (Trower et al., 1990) and the hazard-precaution system (Boyer and Lienard, 2006). In our own work, we have called it the security motivation system (Szechtman and Woody, 2004). Our research investigating this system has focused on its role in everyday circumstances, such as behavior to manage threats of contagion due to dirt and germs, and in pathological variants of these behaviors, such as the compulsive hand-washing seen in obsessive-compulsive disorder (OCD). However, it is likely that the influence of the security motivation system extends well beyond such relatively mundane circumstances. The purpose of this perspective article is to explain briefly what we know about the security motivation system and to advance the following question: Does this biological system affect policy-making about security in important ways? We hope to stimulate the thinking of researchers who investigate security-related decision-making, in particular by sketching some of the kinds of hypotheses that could be examined in such research.

## Properties of the security motivation system

The security motivation system is hypothesized to be a reasonably distinct module in the brain, which evolved to be specially adapted for handling potential threats (Tooby and Cosmides, 1990, 1992, 2006; Trower et al., 1990; Pinker, 1997). Such a module has several key characteristics. First, it is dedicated to the detection of particular types of stimuli as input, rapidly processing a special class of information of particular relevance for survival. Second, when activated, it functions as a motivational system, driving relevant responses (Kavaliers and Choleris, 2001). Third, its output consists of a characteristic set of species-typical behaviors, and engagement in these behaviors plays a crucial role in terminating the activation of the module.

### Type of stimuli that activate the system

Research on how animals manage the threat of predation illuminates the kinds of stimuli that activate the security motivation system. Animals use subtle, indirect cues of uncertain significance as indicators of potential danger (Blanchard and Blanchard, 1988; Lima and Bednekoff, 1999). Evaluating these indirect cues of potential danger is quite different from recognizing imminent danger, such as the actual presence of a predator, and has been characterized in terms of “labile perturbation factors” (Wingfield et al., 1998) and “hidden-risk mechanisms” (Curio, 1993). In short, the security motivation system is tuned to partial, uncertain cues of potential threat, rather than the recognition of imminent danger.

### Nature of activation of the system

Studies of the threat of predation show that relatively weak cues readily activate vigilance and wariness (Brown et al., 1999). In addition, this activation ebbs only slowly (Wingfield et al., 1998), even if no further, confirming cues follow (Masterson and Crawford, 1982; Curio, 1993; Marks and Nesse, 1994). This protracted activation motivates security-related behaviors. In short, weak cues can readily activate the security motivation system, and once activated, it has a protracted half-life and drives behavior.

### Output behaviors and termination of activation of the system

The resulting acts consist of precautionary behaviors, which include probing the environment, checking, and surveillance to gather further information about any potential risks (Blanchard and Blanchard, 1988; Curio, 1993). They also include corrective or prophylactic behaviors, such as washing, that would lessen the effects of the danger if it were to eventuate. Of particular importance, we have characterized security-related behavior as “open-ended,” meaning that the environment does not normally provide a clear terminating stimulus to signal goal attainment (Szechtman and Woody, 2004). For example, if checking does not reveal the presence of a predator, this is not a clear indication of reduced risk (Curio, 1993); that is, the success of precautionary behavior is a non-event. Consequently, we proposed that it is the engagement in security-related behavior in itself that terminates security motivation. In short, activation of the security motivation system elicits precautionary behavior, and the system uses these actions themselves as the terminator of the motivation.

## Neural and physiological basis and empirical evidence for the security motivation system

We have proposed a fairly detailed neuroanatomical-circuit model for the security motivation system, which is based on functional loops consisting of cascades of cortico-striato-pallido-thalamo-cortical connections (Alexander et al., 1986; Brown and Pluck, 2000), with feedback connections from the brainstem to terminate activity in these loops (Szechtman and Woody, 2004; Woody and Szechtman, 2011). We have also described the proposed physiological mechanisms of the security motivation system, which involve regulation of the parasympathetic nervous system and activation of the hypothalamic-pituitary-adrenocortical (HPA) axis (Woody and Szechtman, 2011).

We have demonstrated that activation and subsequent deactivation of the security motivation system can be tracked both with subjective ratings (e.g., anxiety and urge to engage in precautionary behavior) and also physiological changes, especially respiratory sinus arrhythmia (RSA; Porges, 2001, 2007a,b), based on heart-rate variability (Hinds et al., 2010). Using these measures, we have conducted a series of experiments that support the hypotheses that the security motivation system has the aforementioned characteristic properties. First, we have shown that the system is responsive to relatively weak, uncertain cues for potential danger (Hinds et al., 2010, Experiment 1). Second, we have shown that activation of the system, in the absence of subsequent precautionary behavior, is persistent over time, dissipating only very slowly (Hinds et al., 2010, Experiment 2). Third, we have shown that corrective behavior, such as hand washing in response to uncertain cues for contamination, deactivates the system (Hinds et al., 2010, Experiment 1). In contrast to the deactivating effect of corrective behavior, the security motivation system, once it has been activated by uncertain cues, is relatively unresponsive to clear cognitive information that disconfirms the potential threat (Hinds et al., 2010, Experiment 3). This finding supports the hypothesis that the system is action-oriented, and engagement in some kind of precautionary behavior plays a crucial role in turning it off.

In a somewhat parallel series of experiments, we have tested our hypothesis that OCD represents a dysfunction of the security motivation system (Szechtman and Woody, 2004; Woody and Szechtman, 2005). It is well known that the content of OCD revolves around issues of potential danger, such as the threat of contamination or physical harm to oneself or close others (e.g., Reed, 1985; Wise and Rapoport, 1989). We hypothesized that in OCD patients, security motivation is activated in a manner that is reasonably similar to how it is activated in non-patients; however, in OCD patients, subsequent precautionary behaviors fail to turn this activation off in the usual fashion. Thus, once activated, OCD patients remain preoccupied with issues of potential danger for a protracted period of time and repeat the precautionary behaviors over and over again, in an attempt to deactivate the concerns. Our experimental data support this hypothesis that OCD is a stopping, rather than a starting, problem (Hinds et al., 2012). In particular, exposure to uncertain cues for contamination activates the security motivation system similarly in OCD patients and control non-patients, as indexed by both subjective measures and RSA. However, a subsequent fixed period of hand-washing, which returns the non-patients to baseline, has no significant effect on the activation levels of the OCD patients.

## Implications of the security motivation system

The security motivation system would be expected to have some important characteristics that are common to evolved, special-purpose modules. One important characteristic of such modules is that they tend to be encapsulated, operating relatively automatically and autonomously, and their internal computations are not accessible to introspection (Fodor, 1983). That is, they operate largely in the background, apart from the realm of volitionally directed formal logic, and their outputs become evident to the individual intuitively as feelings.

This distinction between a feeling-based system and rational analysis may not always be readily evident in everyday circumstances, because normally the two kinds of output are reasonably well aligned. However, the distinction becomes extremely striking in OCD. OCD patients feel driven to continue their obsessive concerns about potential danger and to repeat precautionary behaviors, such as checking or washing, even though at a rational level they find these concerns and behaviors to be excessive, illogical, and even absurd (Hollander et al., 1996). Indeed, OCD demonstrates that an intuitive, feeling-based module like the security motivation system is very powerful and can override the rational control of behavior.

The relatively automatic, intuitive, feeling-based operation of the security motivation system corresponds with what Kahneman (2011) has termed System 1, in contrast to the formal logic of System 2. What is important to appreciate is that even though the intuitive feelings generated by the security motivation system are vivid, immediate, and phenomenologically compelling to the individual, they are not the same as objective reality, nor are they necessarily closely aligned to conclusions derivable from formal logic. They are, in essence, intuitions that worked well in our remote past but may have limited applicability to any specific, current set of circumstances.

## Does this biological system influence policy-making about security in important ways?

The nature of the security motivation system may have important implications for policy makers wishing to involve others, such as the public, in the detection and appraisal of potential threat, as well as to shape their perceptions and get their support for policy initiatives. Even though the security motivation system is sensitive to the detection of slight, partial, uncertain cues, it evolved in such a way that it is tuned more to certain types of stimuli, but not others. It seems clear that the security motivation system is particularly sensitive to concrete and surprising or novel changes in the environment, and relatively insensitive to relatively abstract and gradual changes (which can become familiar and therefore lack novelty). Thus, for example, hearing some details of the latest terrorist attack, even if it occurred at a distant location, is likely to much more readily elicit activation of the security motivation system than is information about global warming, which is relatively abstract and involves very slow change. In addition, because activation of the security motivation system leads to probing for further information, there is a positive feedback cycle in which further concrete details are added, magnifying the initial difference.

Let's examine the case in which it seems relatively difficult influence others to take potential threats seriously, such as global warming. We would advance the hypothesis that for stimuli to be regarded as possible indicators of potential threat, they must elicit the feeling of a potential threat—that is, anxiety, and wariness, which is the indication that the security motivation system is activated. In other words, if the indicators of a putative potential threat fail to evoke the emotional resonance of potential threats, then the potential threat in question will not be perceived as credible. Because the cues for the potential threat of global warming are abstract, distant, and involve very gradual change, they do not resemble the types of cues the security motivation system is designed to respond to. We would suggest that this is why the issue strikes many people as “academic” or merely political—the relevant cues lack the feeling of potential threats, because they do not readily activate the security motivation system. One solution may be to use the arts to help supply the missing emotion. This is a possibility that is currently being explored in many ways by artists—film-makers, painters, writers, and so on—and directors of art museums; the idea, in the words of a director of New York's Museum of Modern Art, is to “touch and disturb” people and get them engaged (Economist, 2013, July 20–26).

The opposite type of case is one in which stimuli too readily activate the security motivation system, as with some terrorist incidents, in which the attention-grabbing qualities of some potential dangers may have little relation to and even interfere with objective analysis of their severity or likelihood. To inject these more abstract considerations into the operation of the security motivation system requires connecting System 2, which handles abstract ideas, to System 1, which is based on concrete stimuli. We would hypothesize that to be effective, information putting potential threats into a broader critical perspective needs to come early, prior to exposure to the potential-threat stimuli. According to our model of the functional components of the security motivation system (Szechtman and Woody, 2004), such information can come into play at the stage of appraisal of potential danger, which integrates internal factors, such as plans, with external factors, such as concrete stimuli. In contrast, our work suggests that once the security motivation system is activated, it is not affected much by further cognitive information, but instead becomes highly action-oriented, driving, for example, checking and corrective behaviors rather than reappraisal (Hinds et al., 2010).

Of course, the security motivation system theory may have implications for policy-makers themselves, rather than simply those they hope to influence. For everyone, this system is intuitive and feeling-based, operating at least somewhat independently of rational analysis. Because the emotions that the system generates evolved to address crucial survival issues, they are powerful and strongly motivating. Thus, it is natural for decision-makers engaged with an issue of potential danger to be guided by their “gut feelings,” which are more vivid and pressing than the details of rational analysis. Unfortunately, feelings of potential threat (wariness and anxiety) are likely to map imperfectly onto the reality of potential threat. In a related vein, Schneier (2008) pointed out: “Security is both a feeling and a reality. And they are not the same.” A rational analysis of potential danger would need to take account of probabilities and other statistical information, but the intuitive operations of the security motivation system do not work this way. According to Suskind (2006, p. 62), as Vice President, Dick Cheney took the position that potential threats should not be evaluated according to “our analysis, or finding a preponderance of evidence,” but instead by a “one-percent doctrine”: if there is any chance of the reality of the threat, “we have to treat it as a certainty in terms of our response.” This position has a gut-intuitive appeal, in that fragmentary cues suggesting any potential of threat activate security motivation, which in turn naturally drives action. However, this is unlikely to be an adequate basis for making very difficult decisions about how to allocate resources to security-related behavior vs. other important goals.

We would also hypothesize that work circumstances that divide up the tasks involved in managing potential threats may tend to disrupt the stopping mechanism of the security motivation system—because, for example, the policy makers do not get to carry out any of the protective actions themselves. We would propose that this problem can lead some agencies working on security issues to function in a way that is analogous to our characterization of OCD—namely, too much can seem like too little (Hinds et al., 2012). Consider, for instance, that between 2001 and 2013, the Foreign Intelligence Surveillance Act (FISA) court of 14 judges in the USA approved 20,909 requests to monitor individuals or search properties, and turned down only 10. Recently, they apparently ruled that all American phone calls should be considered “relevant” to the investigation of terrorist threats (Economist, 2013, July 13–19). The reason why everything may come to seem relevant may be that the stopping function of the security motivation system is based not on cognitive closure, but instead on concrete action, and those setting policy may not be involved in protective and corrective action at all (e.g., searching and evaluating records).

There are also other implications of the idea that the precautionary behaviors are crucial for turning off security motivation. The security motivation system operates according to what Kahneman (2011) terms System 1 processes. Unfortunately, as Kahneman has very convincingly demonstrated, System 1 is prone to substituting something that has only the form or appearance of a solution in place of a real solution, especially if the better solution would be more difficult. Thus, although turning off the anxiety of security motivation requires action, the details of what is done may not matter as much to the system. Possibly for this reason, policy-making responses to potential threats often seem only to be reactive, rather than proactive. For example, to prevent another shoe-bombing attempt, it is decided that all passengers' shoes must be inspected. Such a prescribed set of actions may be effective in calming security motivation for both policy-makers and the public. However, such a solution seems to ignore the fact that biological agents (even germs) change strategies, so that what would have worked against them in the past may not do so in the future.

## Conclusion

The foregoing hypotheses illustrate just a few of the ways in which the security motivation system theory could be used to generate interesting hypotheses for research on the psychology of security-related policy-making. Although these hypotheses need to be evaluated in future research, we hope they provide a convincing case that the security motivation system theory offers a novel, generative framework for advancing our understanding of policy-making processes related to security and potential danger.

### Conflict of interest statement

The authors declare that the research was conducted in the absence of any commercial or financial relationships that could be construed as a potential conflict of interest.

## References

[B1] Alexander G. E. Delong M. R. Strick P. L. (1986). Parallel organization of functionally segregated circuits linking basal ganglia and cortex. Annu Rev. Neurosci. 9, 357–381 10.1146/annurev.ne.09.030186.002041 3085570

[B2] Blanchard D. C. Blanchard R. J. (1988). Ethoexperimental approaches to the biology of emotion. Annu. Rev. Psychol. 39, 43–68 10.1146/annurev.ps.39.020188.000355 2894198

[B4] Boyer P. Lienard P. (2006). Why ritualized behavior? precaution systems and action parsing in developmental, pathological and cultural rituals. Behav. Brain Sci. 29, 595–613 10.1017/S0140525X06009332 17918647

[B5] Brown J. S. Laundre J. W. Gurung M. (1999). The ecology of fear: optimal foraging, game theory, and trophic interactions. J. Mammal. 80, 385–399 10.2307/1383287

[B6] Brown R. G. Pluck G. (2000). Negative symptoms: the ‘pathology’ of motivation and goal-directed behaviour. Trends Neurosci. 23, 412–417 10.1016/S0166-2236(00)01626-X 10941190

[B8] Curio E. (1993). Proximate and developmental aspects of antipredator behavior. Adv. Study Behav. 22, 135–238 10.1016/S0065-3454(08)60407-6

[B9] Economist. (2013, July 13–19). Surveillance: silence in court. The Economist 408, 8844, p. 29 11154521

[B10] Economist. (2013, July 20–26). Art about climate change: chilling. The Economist 408, 8845, pp. 72–73

[B11] Fodor J. A. (1983). The Modularity of Mind: An Essay on Faculty Psychology. Cambridge, MA: MIT Press

[B12] Hinds A. L. Woody E. Z. Drandic A. Schmidt L. A. Van Ameringen M. Coroneos M. (2010). The psychology of potential threat: properties of the security motivation system. Biol. Psychol. 85, 331–337 10.1016/j.biopsycho.2010.08.003 20723575

[B13] Hinds A. L. Woody E. Z. Van Ameringen M. Schmidt L. A. Szechtman H. (2012). When too much is not enough: obsessive-compulsive disorder as a pathology of stopping, rather than starting. PLoS ONE 7:e30586 10.1371/journal.pone.0030586 22291994 PMC3266914

[B14] Hollander E. Kwon J. H. Stein D. J. Broatch J. Rowland C. T. Himelein C. A. (1996). Obsessive-compulsive and spectrum disorders: overview and quality of life issues. J. Clin. Psychiatry 57 Suppl. 8, 3–6 8698678

[B15] Kahneman D. (2011). Thinking, Fast and Slow. Toronto, ON: Doubleday Canada

[B16] Kavaliers M. Choleris E. (2001). Antipredator responses and defensive behavior: ecological and ethological approaches for the neurosciences. Neurosci. Biobehav. Rev. 25, 577–586 10.1016/S0149-7634(01)00042-2 11801283

[B17] Lima S. L. Bednekoff P. A. (1999). Temporal variation in danger drives antipredator behavior: the predation risk allocation hypothesis. Am. Nat. 153, 649–659 10.1086/303202 29585647

[B18] Marks I. M. Nesse R. M. (1994). Fear and fitness: an evolutionary analysis of anxiety disorders. Ethol. Sociobiol. 15, 247–261 10.1016/0162-3095(94)90002-7

[B19] Masterson F. A. Crawford M. (1982). The defense motivation system - a theory of avoidance-behavior. Behav. Brain Sci. 5, 661–675 10.1017/S0140525X00014114

[B20] Pinker S. (1997). How the Mind Works. New York, NY: Norton 10.1111/j.1749-6632.1999.tb08538.x 10415890

[B21] Porges S. W. (2001). The polyvagal theory: phylogenetic substrates of a social nervous system. Int. J. Psychophysiol. 42, 123–146 10.1016/S0167-8760(01)00162-3 11587772

[B22] Porges S. W. (2007a). A phylogenetic journey through the vague and ambiguous Xth cranial nerve: a commentary on contemporary heart rate variability research. Biol. Psychol. 74, 301–307 10.1016/j.biopsycho.2006.08.007 17055142 PMC1828879

[B23] Porges S. W. (2007b). The polyvagal perspective. Biol. Psychol. 74, 116–143 10.1016/j.biopsycho.2006.06.009 17049418 PMC1868418

[B24] Reed G. F. (1985). Obsessional Experience and Compulsive Behaviour: A Cognitive-Structural Approach. Orlando, FL: Academic Press, Inc

[B25] Schneier B. (2008). The Psychology of Security. Available online at http://www.schneier.com/essay-155.html

[B28] Suskind R. (2006). The One Percent Doctrine. New York, NY: Simon and Schuster

[B29] Szechtman H. Woody E. (2004). Obsessive-compulsive disorder as a disturbance of security motivation. Psychol. Rev. 111, 111–127 10.1037/0033-295X.111.1.111 14756589

[B30] Tooby J. Cosmides L. (1990). The past explains the present: emotional adaptations and the structure of ancestral environments. Ethol. Sociobiol. 11, 375–424 10.1016/0162-3095(90)90017-Z

[B31] Tooby J. Cosmides L. (1992). “Psychological foundations of culture,” in The Adapted Mind: Evolutionary Psychology and the Generation of Culture, eds Barkow J. H. Cosmides L. Tooby J. (New York, NY: Oxford University Press), 19–136

[B32] Tooby J. Cosmides L. (2006). The evolved architecture of hazard management: Risk detection reasoning and the motivational computation of threat magnitudes. Behav. Brain Sci. 29, 631–633 10.1017/S0140525X06009538

[B33] Trower P. Gilbert P. Sherling G. (1990). “Social anxiety, evolution, and self-presentation: an interdisciplinary perspective,” in Handbook of Social and Evaluation Anxiety, ed Leitenberg H. (New York, NY: Plenum Press), 11–45

[B34a] Wingfield J. C. Maney D. L. Breuner C. W. Jacobs J. D. Lynn S. Ramenofsky M. (1998). Ecological bases of hormone–behavior interactions: The “Emergency Life History Stage”. Amer. Zool. 38, 191–206 10.1093/icb/38.1.191

[B34] Wise S. Rapoport J. L. (1989). “Obsessive compulsive disorder - is it a basal ganglia dysfunction?” in Obsessive Compulsive Disorder in Children and Adolescence, ed Rapoport J. (Washington, DC: American Psychiatric Press), 327–344

[B35] Woody E. Z. Szechtman H. (2005). Motivation, time course, and heterogeneity in obsessive-compulsive disorder: response to Taylor, McKay, and Abramowitz (2005). Psychol. Rev. 112, 658–661 10.1037/0033-295X.112.3.658 16060755

[B36] Woody E. Z. Szechtman H. (2011). Adaptation to potential threat: the evolution, neurobiology, and psychopathology of the security motivation system. Neurosci. Biobehav. Rev. 35, 1019–1033 10.1016/j.neubiorev.2010.08.003 20727910

